# Experimental and computational models for intracardiac flow analysis with blood speckle imaging

**DOI:** 10.1371/journal.pone.0349435

**Published:** 2026-05-15

**Authors:** Megan Laughlin, Justin T. Jack, Sam E. Stephens, Elijah Bolin, Paul C. Millett, Morten O. Jensen

**Affiliations:** 1 University of Arkansas, Department of Biomedical Engineering, Fayetteville, Arkansas, United States of America; 2 University of Arkansas, Department of Mechanical Engineering, Fayetteville, Arkansas, United States of America; 3 University of Arkansas for Medical Sciences, Department of Pediatrics, Little Rock, Arkansas, United States of America; 4 Arkansas Children’s Research Institute, Little Rock, Arkansas, United States America; 5 University of Arkansas for Medical Sciences, Department of Surgery, Little Rock, Arkansas, United States of America; NED University of Engineering and Technology, PAKISTAN

## Abstract

Intracardiac flow analysis aims to evaluate blood flow patterns and associated parameters for the assessment of cardiac function. However, there is limited understanding as to how flow parameters are influenced by various sources, such as pressure upstream/downstream and cardiac chamber compliance. The objective of this study was to investigate experimental and computational tissue-mimicking models to be used alongside 2D Blood Speckle Imaging for intracardiac flow analysis. Two geometries, an axisymmetric swell and idealized left ventricle, were utilized. As an initial parameter of interest, the pressure-drop across each geometry was determined from tissue-mimicking phantoms using direct pressure measurements, blood speckle imaging, and 3D computational fluid dynamics simulations with fluid-structure interaction. The results indicate limited quantitative agreement between direct measurements, 2D blood speckle imaging, and 3D computational fluid dynamics, with qualitative agreement capturing a consistent shape of the pressure drop curve between methods. Additionally, the importance of phantom design is demonstrated due to the likely impact of gel thickness on flow patterns and their associated measurements. The findings of this study indicate that future work focusing on the optimization of BSI settings and increasing model complexity with the inclusion of cardiac valves and patient-specific geometries are still required. These models may then allow for further tuning of variables to better understand their effect on various intracardiac flow parameters, and ultimately their clinical applicability.

## Introduction

Ultrasound-based assessment of left ventricular (LV) function has traditionally relied on changes in LV size between systole and diastole; some of the most common measures include shortening and ejection fractions [1]. Nevertheless, these parameters do not account for the potential impact of intracardiac flow disturbances on myocardial function. Recently, intracardiac flow analysis as a marker of LV function has gained traction [2,3]. Of particular interest are vortices, a signature flow feature within the LV that aid in the preservation of kinetic energy and ensure a smooth redirection of blood into the left ventricular outflow tract [4,5]. Vortices are inherently unstable fluid structures, and their disruption leads to irregular flow patterns that have been linked to cardiac dysfunction [6,7]. However, a wide range of parameters related to these vortices have been utilized to assess intraventricular flow with limited information on how such parameters may be influenced by changes from various sources.

Flow data can be acquired using various imaging modalities, including echocardiography methods, such as 2D vector flow mapping and particle image velocimetry, and 4D flow cardiac magnetic resonance imaging [8–10]. This presents additional complexity as changes in intraventricular flow parameters may not be equivalent when quantified using modalities of differing spatial dimensions. These gaps in knowledge and a lack of standardization have limited the clinical use of intracardiac flow analysis.

Experimental and computational models allow for manipulation of variables, such as LV compliance and pressure upstream/downstream, to evaluate their effects on flow patterns. Both the fluid and solid components of these models are important considerations to best simulate the physiological environment. Previous studies have utilized *in vitro* flow phantoms from tissue-mimicking materials that exhibit the mechanical, acoustic, and/or optical properties of cardiovascular tissues with a blood mimicking fluid (BMF) [11,12]. The BMF is compatible with ultrasound-based vector imaging modalities, which is the preferred clinical imaging method due to its noninvasiveness.

Fluid-Structure Interaction (FSI) models incorporated in Computational Fluid Dynamics (CFD) simulations can predict the complex coupling of a deformable solid and the surrounding fluid [13,14]. This provides greater understanding of the interactions between solid mechanics and fluid dynamics within the heart, and how they relate to overall cardiac function. The use of FSI for cardiovascular applications is rapidly growing with recent work utilizing these models to simulate intracardiac flow for idealized and patient-specific cases [15–17]. The predictive power of FSI models without direct physical validation is, however, limited.

The objective of this work serves as an exploratory investigation of experimental and computational models to be used alongside Blood Speckle Imaging (BSI) for intracardiac flow analysis. BSI is a 2D flow imaging modality combining high-frame rate ultrasound with speckle tracking to directly quantify and visualize blood flow [18–20]. For both experimental and computational models, *in vitro* phantoms were used with a tissue-mimicking gel and BMF as the solid and fluid, respectively. Pressure-drop across the phantom geometry was measured directly and using velocity-field data from both BSI and CFD as an initial parameter of interest due to its widespread clinical use in the evaluation of congenital heart defects. Within this framework, numerical verification of the CFD framework—including mesh and timestep convergence and analytical benchmarks—is distinguished from experimental validation, with the latter being limited by the extent of the available in vitro measurements. The overall goal was to perform relative comparisons to establish a relationship between direct pressure, 2D BSI, and 3D CFD measurements from experimental and computational tissue-mimicking models. These models may then be employed to better understand the effects of changes in various sources on flow parameters and thus their usefulness in intracardiac flow analysis. The subsequent sections describe the experimental setup for the *in vitro* phantom and flow loop system along with data acquisition protocols for direct pressure measurements and BSI, followed by details of the 3D FSI numerical simulations. Analysis of pressure drop and velocity magnitudes across these methods are reported and discussed.

## Materials and methods

### In vitro phantoms

**Fig 1:**
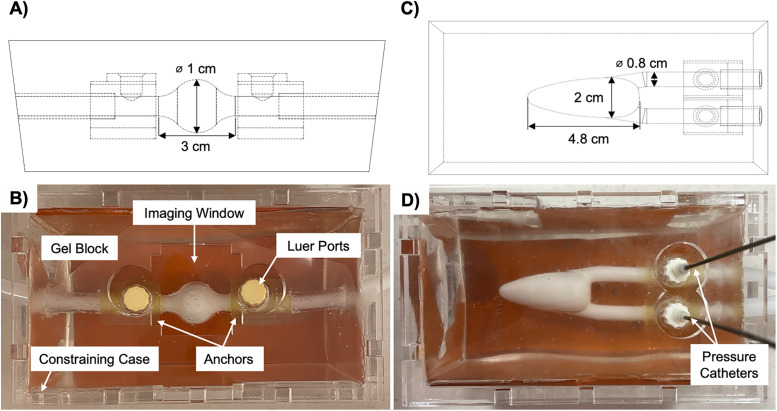
Phantom models of the (A-B) swell and (C-D) idealized left ventricle geometries for experimental and computational use. The *in vitro* tissue-mimicking phantoms demonstrate the (B) constraining case with imaging windows used for Blood Speckle Imaging (BSI) and (D) pressure catheters at the inlet and outlet for direct pressure measurements.

Swell and idealized LV geometries were modeled in SolidWorks (Dassault Systèmes SolidWorks Corporation, Waltham, MA) (Fig 1A and 1C). BSI is currently limited to pediatric cardiac applications due to its relatively shallow penetration depth, therefore the LV model dimensions were based on the 50^th^ percentile length and weight for a six-month old [21,22]. Cardiac valves were omitted from the models to minimize the variables impacting fluid flow.

Tissue-mimicking phantoms were fabricated using a medical gel (Gel 1, Humimic Medical, LLC, Greenville, SC) that best simulates human myocardium per a previously reported method [12]. The phantoms consisted of a gel block with a void corresponding to the geometry of interest (Fig 1B and 1D). To investigate the impact of gel thickness and symmetry on flow and the resulting pressure-drop, three phantoms were made for each geometry that varied by depth within the gel. Phantoms were labeled as Depth 1−3, with Depth 2 located directly in the middle of the gel block, and Depth 1 and Depth 3 displaced −0.5 cm and 0.5 cm, respectively.

An acrylic case with imaging windows sized to fit the ultrasound transducer was fabricated to provide phantom stabilization and minimize unwanted movement capable of impacting *in vitro* measurements. Silicone rubber sheets (Norseal 9030, Saint-Gobain, Solon, OH) were placed between the phantom and case opposite the windows to act as acoustic absorbers and prevent echoes from the case during BSI [23].

**Fig 2:**
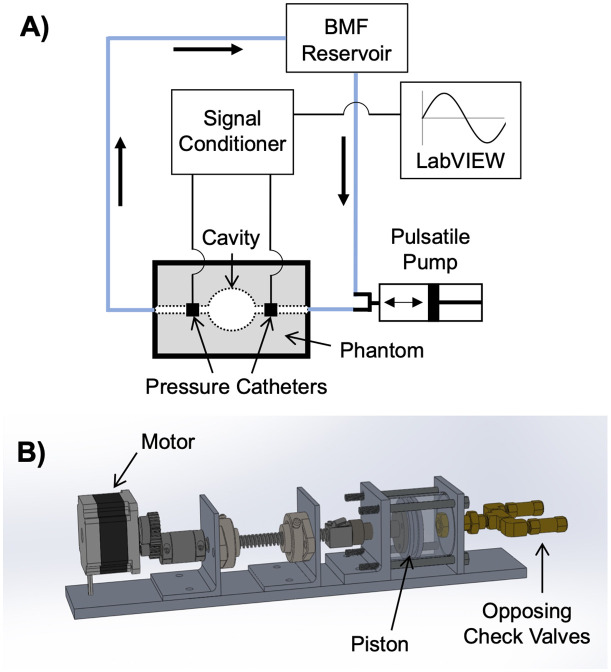
Experimental setup. **(A)** Diagram of the experimental *in vitro* flow loop set-up used for direct pressure measurements and Blood Speckle Imaging (BSI) of the phantoms. A blood-mimicking fluid (BMF) was circulated by **(B)** a custom pulsatile pump.

An *in vitro* flow loop provided pulsatile flow of a BMF (Shelley Medical Imaging Technologies, Ontario, Canada) to the phantoms using a custom-built pulsatile pump (Fig 2) programmed to simulate the cardiac output, stroke volume, and LV ejection time for infants [24–27]. The flow waveform consisted of a square wave with sine-like ramp up/down, flowrate of approximately 2.5 L/min and total pulse duration of 1.7s, including a 0.2s flow pulse and 1.5s delay between pulses to allow for gel relaxation and avoid overexpansion.

### Direct pressure measurements

Mikro-Tip™ pressure catheters (SPR 350-S, Millar, Inc., Houston, TX) were connected to the phantoms before and after the geometry of interest (Fig 2A). Data was acquired in LabVIEW using a USB-6009 DAQ (National Instruments, Austin, TX) and two-channel pressure control unit (PCU-2000, Millar, Inc.).

Pressure data was recorded for six flow pulses for each phantom, with the first three omitted from all analyses to ensure the establishment of flow and pressure. The following three pulses were averaged together to produce representative pressure curves for the inlet and outlet. Pressure-drop (ΔP) was subsequently calculated by:


$$\begin{array}{c} \Delta P=P_{inlet}-P_{outlet} \end{array}$$ (1)

### 2D Blood speckle imaging

BSI was performed using a Vivid E95 with BSI software (GE Vingmed Ultrasound, Horten, Norway) and 6MHz phased-array probe (6S, GE Healthcare, Milwaukee, WI). Scans were obtained from the side of each swell (n = 4) and LV (n = 2 for Depth 1, n = 4 for Depths 2–3) phantom. All settings were optimized to achieve sufficient signal within the void and minimize artifacts, resulting in frame rates of 236–298 frames/sec. BSI data was processed using research-only software to obtain the velocity-fields and exported for offline processing in MATLAB (The Mathworks Inc., Natick, MA).

Tracking quality (TQ) is a characteristic of the velocity-field data ranging from 0 (low) to 1 (high). A good correlation between TQ and the accuracy of velocity measurements has been reported for a threshold of 0.4 and therefore only velocities with a TQ > 0.4 were used for analysis [20].

Pressure-drop from BSI was calculated from the velocity field. For an incompressible fluid and neglecting external forces, the Navier-Stokes equations can be written as:


$$\begin{array}{c} \rho \left(\frac{\partial u}{\partial t}+u\cdot \nabla u\right)=-\nabla P+\mu \nabla^{\text{2}}u \end{array}$$ (2)

$$\begin{array}{c} \nabla \cdot u=\text{0} \end{array}$$ (3)

where u = velocity vector, t = time, ρ = density, and μ = dynamic viscosity [28]. For inviscid flow, Eq 2 can be simplified to obtain the pressure gradient in each spatial dimension:


$$\begin{array}{c} \frac{\partial P}{\partial x,y}=-\rho \left(\frac{\partial u_{x,y}}{\partial t}+u_{x}\frac{\partial u_{x,y}}{\partial x}+u_{y}\frac{\partial u_{x,y}}{\partial y}\right) \end{array}$$ (4)

The pressure-drop (ΔP) can then be calculated between two points along a streamline:


$$\begin{array}{c} \Delta P=\int_{b}^{a}\frac{\partial P}{\partial x}dx+\int_{b}^{a}\frac{\partial P}{\partial y}dy \end{array}$$ (5)

where a = (x_1_,y_1_) at the inlet and b = (x_2_,y_2_) at the outlet which were kept consistent across all timepoints for a single scan [29,30].

### 3D computational fluid dynamics

CFD simulations were conducted in COMSOL Multiphysics© (Version v5.3a) using the Finite-Element Method to solve Eq 2 and Eq 3 for fluid motion and the Arbitrary Lagrangian Eulerian (ALE) method to determine the time-dependent motion of the phantom wall. A two-way, fully coupled FSI approach was adopted for modelling the system. For continuity:


$$\begin{array}{c} \Gamma_{f}\cdot n=\Gamma_{s}\cdot n \end{array}$$ (6)

where ***n*** = vector normal of the fluid-structure interface, $\Gamma_{f}$ = Cauchy stress tensor for the fluid domain and $\Gamma_{s}$ = solid Cauchy stress tensor. A no-slip condition was assumed at the wall of the fluid domain. The top of the tissue-mimicking gel domain was modeled as a free boundary. All remaining exterior gel boundaries were treated as fixed, including the inlet and outlet of the fluid boundary.

To determine the inlet and outlet boundary conditions, a smooth curve sampled from the direct pressure measurement was prescribed as the outlet boundary condition. Next, a similar smooth pressure curve sampled from the inlet direct pressure measurement was utilized to solve for the corresponding flow curve, which was applied as a fully developed inlet boundary condition with the use of weak contributions and a discrete algebraic equation. Initial simulations of flow through a rigid tube were performed to benchmark this flow generated, pressure coupling approach against the theoretical framework by Womersley [31]. The pressure drop was reproduced within 0.5%. Since the Womersley derivation is pressure driven and allows the velocity profile to be unconstrained, there were some differences in the maximum and minimum flow rates, as we prescribed a fully developed inlet boundary condition. Yet, this constraint on the velocity profile improves numerical stability and has been adapted by studies involving pulsatile flow [32,33].

Models for the simulations consisted of four domains: the tissue-mimicking gel, swell or LV (fluid), anchors, and tubing. A summary of solid and fluid properties used are given in Table 1 [34,35]. The tissue-mimicking gel was simulated using a Yeoh hyperelastic model per the best fit of previously obtained mechanical data to various material models [36,37]. Assuming isotropy and incompressibility, this approximates the first Piola-Kirchhoff stress (T) under uniaxial loading as:

**Table 1:** A summary of solid and fluid properties used for the Computational Fluid Dynamics (CFD) simulations.

	Solid	Fluid
**Material**	Tissue-mimicking gel	Blood-mimicking fluid (BMF)
**Density (kg/m^3^)**	937	1037
**Viscosity (mPa ⋅ s)**	–	4.1
**Poisson’s Ratio**	0.49	–
**Other**	Yeoh Model	Newtonian


$$\begin{array}{c} T=\text{2}\left(\lambda -\lambda^{-\text{2}}\right)[c_{\text{1}}+\text{2}c_{\text{2}}\left(I_{\text{1}}-\text{3}\right)+\text{3}c_{\text{3}}{\left(I_{\text{1}}-\text{3}\right)}^{\text{2}}] \end{array}$$ (7)

where λ = principal stretch, $I_{\text{1}}$ = isochoric strain invariant and $c_{\text{1}}$, $c_{\text{2}}$, and $c_{\text{3}}$ = Yeoh material parameters, which were computed to be 7.3kPa, 9.4kPa and 0kPa, respectively. Additionally, a simplified Kelvin-Voight viscoelastic model was implemented, which describes the material dynamics with the governing equations relating stress (σ), strain (ε), and strain rate ($\dot{\varepsilon }$) as:


$$\begin{array}{c} \sigma =E\varepsilon +\eta \dot{\varepsilon } \end{array}$$ (8)

where E = elastic modulus and η = viscosity of the gel. The retardation time (τ) is defined as τ=η/E and was approximated as 0.01s.

Temporal discretization in all simulations was performed using an adaptive backward difference formula (BDF) scheme. Initial simulations were ran allowing a maximum BDF order of five, resulting in fluctuations of a BDF order between one and two. Thus, a maximum BDF order of two was enforced for subsequent runs and the timestep was restricted to a maximum of 5ms. Both timestep and order were automatically reduced when required to ensure stability and that the numerical solution satisfied a relative error tolerance of 1e-4. A fully coupled approach was used to solve the full system of equations using the multifrontal massively parallel sparse direct solver (MUMPS). A relative mass balance check was performed manually in COMSOL. As the simulation implements ALE for the deforming fluid domain, to account for mass accumulation in the deforming fluid domain, the relative mass flow was computed as the net accumulated mass plus the net mass out minus the net mass in, all divided by the net mass out across a cycle (i.e., 1.7s and 2.7s for the swell and LV models respectively). This relative check was also below the relative tolerance of 1e-4.

**Fig 3:**
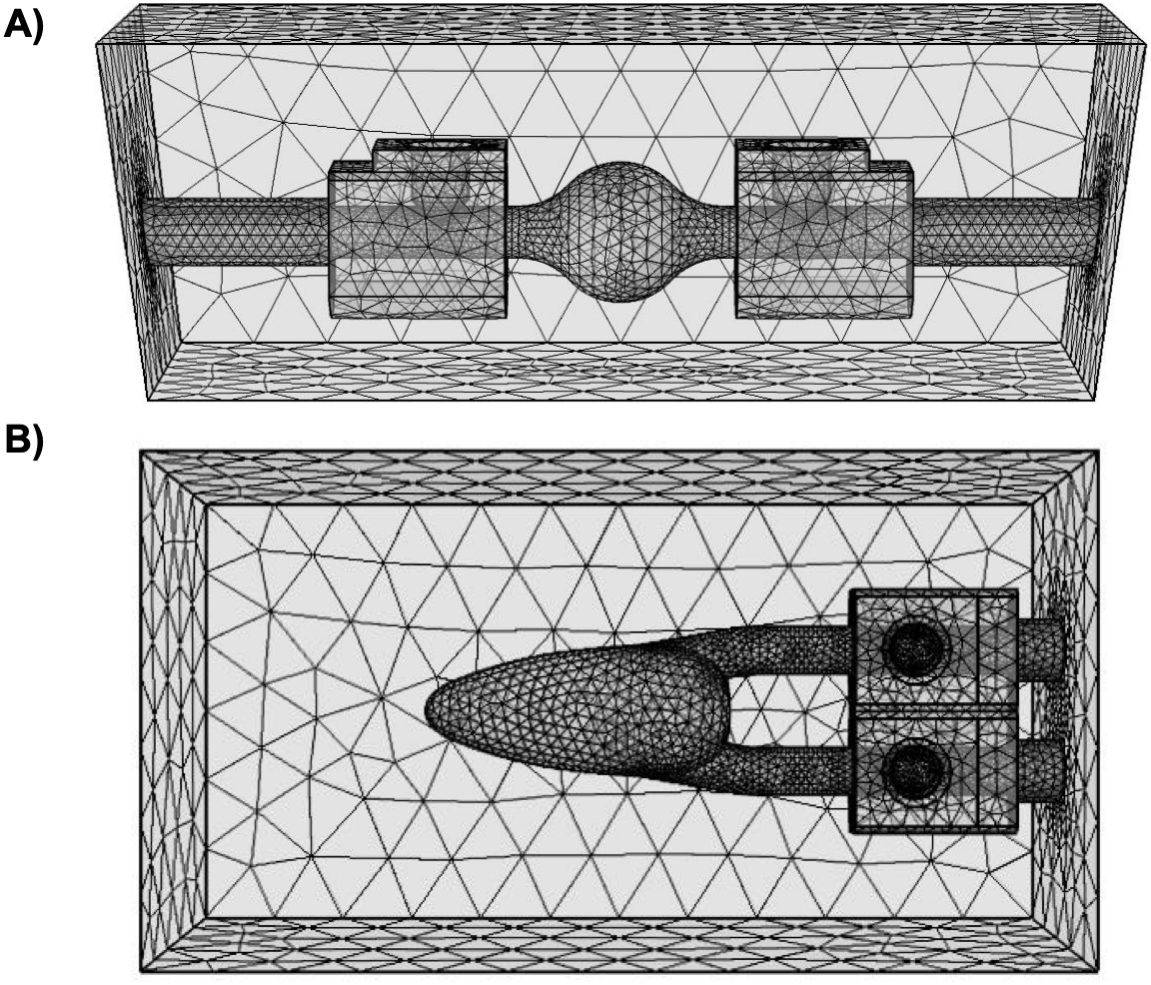
Sample computational meshes. Examples of meshes for the CFD simulations of the **(A)** swell and **(B)** LV phantoms.

The fluid domain utilized COMSOL’s fine mesh calibrated for fluid dynamics with a finer mesh at the fluid-gel interface, and the gel domain used a general physics, fine tetrahedral mesh (Fig 3). Tetrahedral elements were used for all domains. The only exception were prism elements used at the walls of the fluid domain to define the boundary layer. For the fluid domain discretization, linear elements were used for the velocity components and the pressure field. Quadratic elements were used for the discretization of the displacement field of the gel domain. The production mesh for the swell geometries consisted of 111696, 105520 and 105108 mesh elements for depth 1, depth 2 and depth 3 respectively. Similarly, the LV geometries were resolved with 112032, 112288 and 112681 mesh elements for depth 1, depth 2 and depth 3 respectively. A mesh convergence study was performed for depth 1 of the swell geometry. A coarser mesh consisting of 51849 elements and a finer mesh consisting of 202502 elements were simulated in addition to the production mesh. A relative error of less than 1% was computed when comparing the maximum fluid pressure and stroke volume between the production mesh and the coarser mesh. Similarly, a relative error of less than 1% was computed when comparing the maximum fluid pressure and stroke volume between the production mesh and the finer mesh.

## Results

### Direct measurements

**Fig 4:**
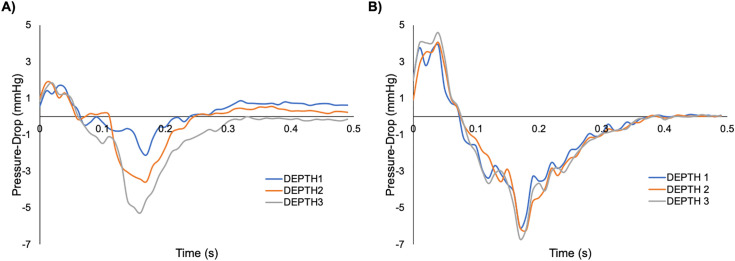
Comparison of direct pressure measurements taken via pressure catheters. **(A)** Three swell phantoms, Depths 1-3, and **(B)** three LV phantoms, Depths 1-3. The curves exhibit the same shape with significant differences observed between the minimum pressure-drops of the swell phantoms (p < 0.01) and maximum pressure-drops of the LV phantoms (p < 0.01).

The maximum and minimum pressure-drops obtained from the swell and LV phantoms with all three methods are summarized in Tables 2 and 3. From direct measurements, a significant difference in the minimum pressure-drops between the swell phantoms was observed (p < 0.01). The pressure-drop curves for all three phantoms followed the same shape with an increase at the beginning of the flow pulse before taking a sharp decrease to a negative pressure-drop (Fig 4A).

**Table 2:** Summary of data for the swell geometry.

Parameter	Method	Depth 1	Depth 2	Depth 3
**Velocity (m/s)**	BSI	1.13 ± 0.19	1.22 ± 0.17	1.40 ± 0.05
	CFD	1.45	1.54	1.43
**Max. Pressure-Drop (mmHg)**	Direct	2.15 ± 0.17	2.32 ± 0.30	2.33 ± 0.36
	BSI	5.50 ± 2.84	2.81 ± 0.77	4.33 ± 1.52
	CFD	1.73	1.91	1.81
**Min. Pressure-Drop (mmHg)**	Direct	−3.43 ± 0.74*^†^	−4.49 ± 0.27*	−5.89 ± 0.22*
	BSI	−8.22 ± 2.32^†^	−6.95 ± 3.63	−5.16 ± 1.31
	CFD	−2.10	−3.62	−5.19

Peak velocity and maximum/minimum pressure-drops for the swell phantoms as determined by 2D Blood Speckle Imaging (BSI), 3D Computational Fluid Dynamics (CFD), and direct pressure measurements. Each phantom differs in depth of the swell geometry void by 0.5 cm.

*p < 0.05 for Depths 1–3.

†p < 0.05 for direct pressure vs. BSI.

**Table 3:** Summary of data for the left ventricle geometry.

Parameter	Method	Depth 1	Depth 2	Depth 3
**Velocity (m/s)**	BSI	1.10 ± 0.04	1.18 ± 0.13	1.10 ± 0.07
	CFD	1.55	1.64	1.69
**Max. Pressure-Drop (mmHg)**	Direct	4.53 ± 0.04*^†^	4.41 ± 0.22*	5.06 ± 0.16*^†^
	BSI	3.49 ± 0.55^†^	3.18 ± 1.64	3.05 ± 0.93^†^
	CFD	3.88	4.01	4.60
**Min. Pressure-Drop (mmHg)**	Direct	−6.69 ± 0.48^†^	−7.09 ± 0.36^†^	−7.55 ± 0.15^†^
	BSI	−2.22 ± 0.67^†^	−0.73 ± 0.49^†^	−1.96 ± 0.90^†^
	CFD	−6.16	−6.44	−6.78

Peak velocity and maximum/minimum pressure-drops for the idealized left ventricle (LV) phantoms. Each phantom differs in depth of the LV geometry void by 0.5 cm.

*p < 0.05 for Depths 1–3

†p < 0.05 for direct pressure vs. BSI.

The pressure-drop curves for the LV phantoms exhibited a similar shape to those of the swell phantoms, albeit with a larger pressure-drop at the beginning of the flow pulse and more negative pressure-drop following (see Fig 4B). The maximum pressure-drops exhibited a significant difference between the three phantoms (p < 0.01).

### BSI

**Fig 5:**
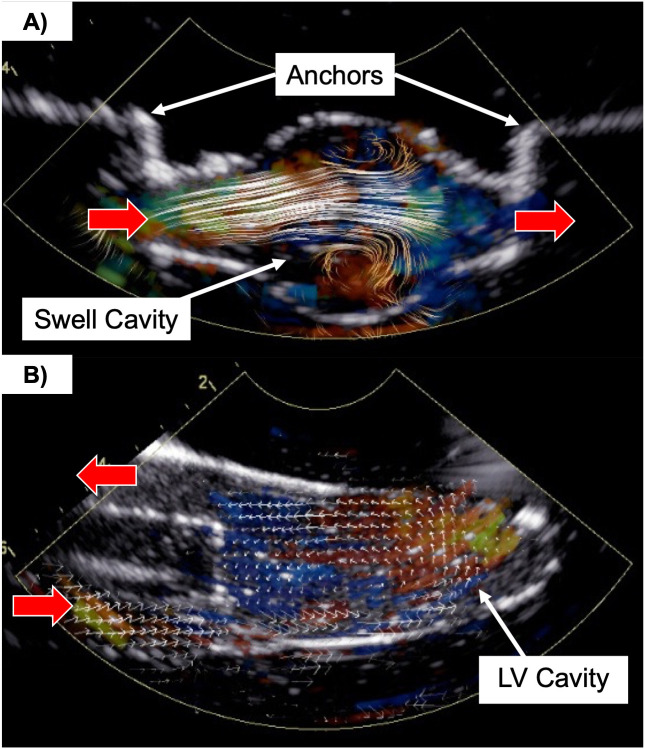
Representation of Blood Speckle Imaging (BSI). **(A)** A swell phantom (Depth 3) during peak flow. Vortices are observed within the upper and lower portions of the swell cavity. **(B)** BSI of a left ventricle (LV) phantom (Depth 2). Flow enters through the inlet (bottom) and forms a vortex within the LV cavity as flow is redirected toward the outlet.

At peak flow, lateral flow is seen entering from the inlet into the swell where vortices form in the upper and lower areas of the cavity (Fig 5A). No significant difference in the maximum or minimum pressure-drops measured by BSI was observed between the swell phantoms (p = 0.19 and p = 0.30, respectively) (Table 2).

In the LV phantom, unidirectional flow is observed through the inlet and along the cavity edge as it curls into a vortex within the cavity to redirect flow toward the outlet (Fig 5B). No significant difference was observed between the maximum or minimum pressure-drops from the LV phantoms using BSI (p = 0.92 and p = 0.07, respectively) (Table 3).

### CFD

**Fig 6:**
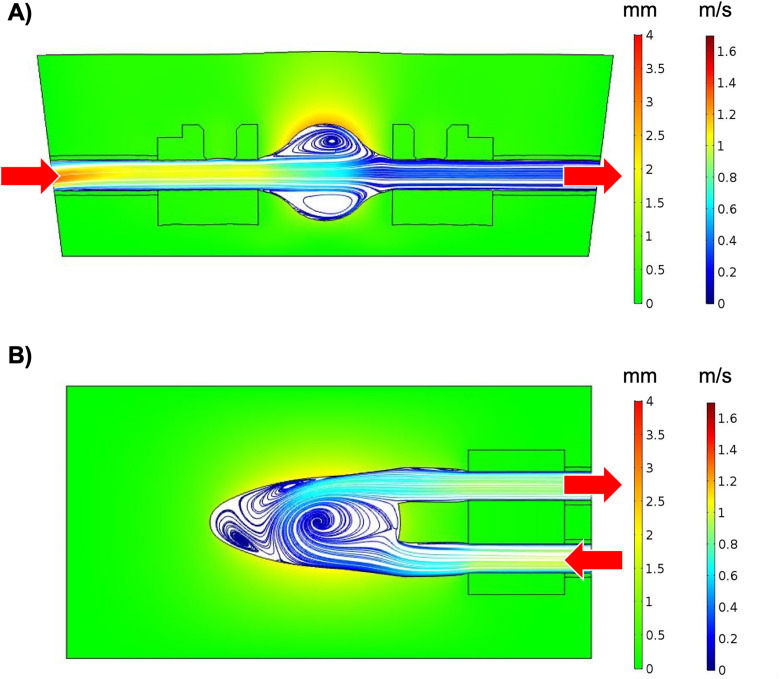
Representation of Computational Fluid Dynamics (CFD). Velocities and displacements from CFD of a **(A)** swell phantom (Depth 1) and **(B)** LV phantom (Depth 1) at peak flow.

At peak flow, flow is seen entering the phantom at high velocities and decreasing in velocity within the swell (Fig 6A). Higher phantom displacements are observed around the upper area of the swell and vortices are present in both the upper and lower portions of the cavity.

More uniform phantom displacements are observed around the LV cavity (Fig 6B). A main vortex is seen redirecting flow toward the outlet at higher velocities while a secondary vortex with low velocities is present within the apex of the cavity.

### BSI/CFD/Direct pressure relationship

**Fig 7:**
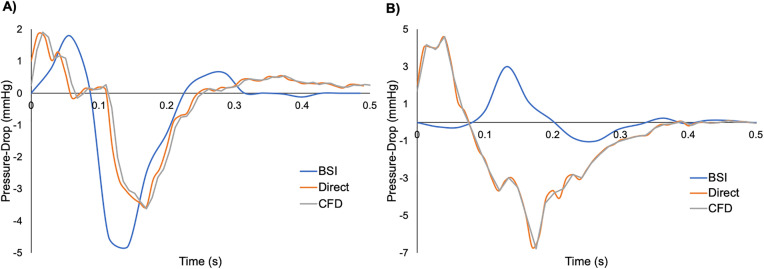
Comparison between pressure drop acquired via the three methods. Pressure-drop curves from **(A)** a swell phantom (Depth 2) and **(B)** a LV phantom (Depth 3) obtained from direct pressure measurements, 2D Blood-Speckle Imaging (BSI), and 3D Computational Fluid Dynamics (CFD).

The maximum velocities measured by BSI were lower than those from CFD for all three phantoms of both the swell (Table 2) and LV (Table 3). An example of pressure-drop curves obtained from all three measurements are given in Fig 7 for both swell and LV phantoms. All three swell curves exhibited similar shapes with an increase in pressure-drop followed by a more drastic decrease to a negative pressure-drop (Fig 7A). The timing of the curves was also consistent within approximately 0.05s and demonstrated agreement of the maximum pressure-drop, but a lower minimum pressure-drop.

The pressure-drop curves from direct measurements and CFD of the LV phantom were indiscernible from one another, while that from BSI exhibited a shift in timing of approximately 0.13s with a lower maximum pressure-drop (Fig 7B). All LVs exhibited an initial increase in pressure-drop followed by a decrease to a negative pressure-drop, but the minimum pressure-drop measured by BSI was apparently higher.

For the swell phantoms, no significant difference in the maximum pressure-drop was observed between direct measurements and BSI (p = 0.10, p = 0.36, and p = 0.08 for Depths 1–3, respectively). Additionally, no significant difference in the minimum pressure-drop measured directly and with BSI was observed for Depth 2 (p = 0.31) or Depth 3 (p = 0.39), while there was a significant difference for Depth 1 (p = 0.02). All pressure-drops from CFD were outside the range of standard deviations (SDs) from direct measurements and BSI for all three depths, except the minimum pressure-drop range from BSI of Depth 3.

No significant difference was present between the maximum pressure-drop from direct and BSI measurements of the Depth 2 (p = 0.26) LV phantom. There was a significant difference in the maximum pressure-drop measured directly and with BSI for Depth 1 (p < 0.01) and Depth 3 (p = 0.01). For minimum pressure-drops, there was a significant difference between direct and BSI measurements for the three LV phantoms (p < 0.01). While the maximum pressure-drops obtained from CFD were within the range for BSI of the Depth 1 and Depth 2 LV phantoms, all other pressure-drops from CFD were outside the range of direct and BSI measurements.

## Discussion

This study indicates limited agreement between direct pressure measurements, 2D BSI, and 3D CFD from experimental and computational models of tissue-mimicking phantoms based upon pressure-drop as an initial parameter of interest. The higher SDs observed for BSI and low sample sizes make it difficult to form conclusions based solely on statistical significance between direct measurements and BSI. This also limits the evaluation of agreement between BSI and CFD due to its reliance upon the pressure-drop range given by the SDs from BSI. The scope of this study should be interpreted within the context of a comparative and exploratory investigation. While numerical verification was performed to improve consistency and stability of the CFD simulations, experimental validation is necessarily limited to direct inlet and outlet pressure measurements. Therefore, the overall trend of the results and more qualitative comparisons such as the shape of the pressure-drop curves is valuable.

Phantom depth was determined to likely effect flow parameters, supported by the significant difference in either the maximum or minimum pressure-drops from direct measurements of the swell and LV phantoms. These differences are also apparent in the pressure-drop curves from the swell phantoms where they exhibit the same shape, but the Depth 2 curve falls in between Depths 1 and 3 for most of the flow pulse. The maximum pressure-drop results indicate Depth 2 having the best agreement between direct measurements, BSI, and CFD. These collective results indicate that gel thickness and overall phantom design are important considerations for models to be used in intracardiac flow analysis due to their potential impact on measured flow parameters. Additionally, the consistently lower velocities observed from BSI vs. CFD suggest the possibility of dimensional differences between the two geometries that may occur during phantom fabrication, but further investigation is necessary.

The swell phantoms demonstrated greater agreement between direct measurements, BSI, and CFD compared to the LV phantoms. This is most evident by comparing the three pressure-drop curves where both the timing and maximum/minimum pressure-drops are more comparable for the swell phantoms.

The LV phantoms presented more difficulty with BSI due to a lack of simultaneous signal at the inlet and outlet. This likely led to inaccuracies in pressure-drops at time points where signal was absent at one location and may explain the higher minimum pressure-drops from BSI. Potential remedies include introducing an angle between the transducer and phantom or adjustment of BSI settings. Additionally, the time-shift for the pressure-drop curve of the LV obtained from BSI (Fig 7B) compared to direct measurements and CFD is likely attributed to signal lag at the outlet influenced by complexity of the phantom geometry. This could be described by the Womersley number, which expresses the phase lag between flow rate and pressure gradient [38,39]. As BSI utilizes velocity data to derive pressure-drop, this may need to be accounted for when comparing those obtained from direct measurements and CFD.

### Study limitations

A limitation of this work is the BSI settings required to optimize signal in the cavity while minimizing signal artifacts outside. The BSI technology utilized has a minimum detectable velocity. While the threshold for this low velocity rejection was somewhat setting dependent, it was not possible to eliminate, to balance signal optimality while minimizing signal artifacts. The pulse repetition frequency (PRF) to achieve this was 8kHz and 4.5kHz for the swell and LV phantoms, respectively. This resulted in rejection of velocities below 21 cm/s for the swell and 13 cm/s for LV phantoms, and therefore the absence of signal in some areas during portions of the flow cycle. A PRF of 5-7kHz has been reported for *in vivo* BSI and could serve as a target range for *in vitro* BSI [20].

The absence of low velocities may provide inaccurate flow analysis due to no signal where flow is present. This prompted the decision to implement the inviscid Navier-Stokes equations to compute the pressure drop along a path over methods like the unsteady Bernoulli equation which are more likely to be affected by the low velocity reject setting [30]. Therefore, it is desired to further optimize the experimental set-up to allow for detection of lower velocities. A reduction in PRF alone would achieve this, but also increases the chance of artifacts. A multipronged approach addressing the tissue-mimicking phantom, BMF, and BSI settings would likely prove most effective. The comparison of velocity-fields from BSI and CFD should also be investigated to provide greater understanding on the agreement between velocity-field data vs. specific parameters derived from that data, such as pressure-drop, but was beyond the scope of this work.

No cardiac valves were included in either model to isolate the relationship between direct measurements and those obtained with BSI and CFD without the complexity of valves. For example, FSI models have demonstrated that mitral valve geometry and leaflet dynamics both influence LV flow and vortex formation [40,41]. Similar findings have been reported for mechanical heart valves [42,43]. The presented work will serve as a foundation to develop more complex models that include mitral and aortic valves for intracardiac flow analysis.

Given the discussed limitations and simplicity of geometries investigated, this work primarily supports indications regarding the use of experimental and computational tissue-mimicking models in intracardiac flow analysis. However, further improvements for experimental measurements along with additional model-specific validation of CFD simulations, and full velocity field comparisons are necessary to capture a more generalize relationship between direct, CFD and BSI data. Additional future work will focus on increased sample sizes and model complexity, including patient-specific geometries. These models may then allow for the manipulation of variables to evaluate the effects on intracardiac flow patterns and associated parameters, including potential differences when quantified with 2D vs. 3D methods. This information will serve to identify the clinical applicability of various intracardiac flow parameters and efficacy of a 2D imaging modality such as BSI in quantifying inherently 3D blood flow.
